# Psychosocial Health Interventions by Social Robots: Systematic Review of Randomized Controlled Trials

**DOI:** 10.2196/13203

**Published:** 2019-05-10

**Authors:** Nicole Lee Robinson, Timothy Vaughan Cottier, David John Kavanagh

**Affiliations:** 1 Australian Centre for Robotic Vision Brisbane Australia; 2 Queensland University of Technology Brisbane Australia

**Keywords:** social robot, healthcare, treatment, therapy, autism spectrum disorder, dementia

## Abstract

**Background:**

Social robots that can communicate and interact with people offer exciting opportunities for improved health care access and outcomes. However, evidence from randomized controlled trials (RCTs) on health or well-being outcomes has not yet been clearly synthesized across all health domains where social robots have been tested.

**Objective:**

This study aimed to undertake a systematic review examining current evidence from RCTs on the effects of psychosocial interventions by social robots on health or well-being.

**Methods:**

Medline, PsycInfo, ScienceDirect, Scopus, and Engineering Village searches across all years in the English language were conducted and supplemented by forward and backward searches. The included papers reported RCTs that assessed changes in health or well-being from interactions with a social robot across at least 2 measurement occasions.

**Results:**

Out of 408 extracted records, 27 trials met the inclusion criteria: 6 in child health or well-being, 9 in children with autism spectrum disorder, and 12 with older adults. No trials on adolescents, young adults, or other problem areas were identified, and no studies had interventions where robots spontaneously modified verbal responses based on speech by participants. Most trials were small (total N=5 to 415; median=34), only 6 (22%) reported any follow-up outcomes (2 to 12 weeks; median=3.5) and a single-blind assessment was reported in 8 (31%). More recent trials tended to have greater methodological quality. All papers reported some positive outcomes from robotic interventions, although most trials had some measures that showed no difference or favored alternate treatments.

**Conclusions:**

Controlled research on social robots is at an early stage, as is the current range of their applications to health care. Research on social robot interventions in clinical and health settings needs to transition from exploratory investigations to include large-scale controlled trials with sophisticated methodology, to increase confidence in their efficacy.

## Introduction

### Background

In recent years, we have seen exciting developments in the application of robotics to medical treatments. Medical robot–assisted surgery in operating theaters enhances patient outcomes of surgical procedures in orthopedics, radiosurgery, and neurology [1-6]. Exoskeleton devices work to enhance strength or improve movement for patients suffering from traumatic brain and spinal cord injury, disability such as stroke and multiple sclerosis, and rehabilitation treatment [7-9]. Surgical and rehabilitative robotics offer distinct advances in their exceptional ability to augment treatment practices to enhance patient outcomes but are restricted to a highly specific field of medical assistance. This leaves other health care services untouched by the potential benefits that robotics may offer for health care professionals and their patients, including psychosocial interventions for health or well-being.

At first thought, robotics for psychosocial interventions may seem counterintuitive. If, as has been argued, the therapeutic relationship is key to positive treatment outcomes [10], how could a robot perform such a task? However, there are precedents for such a role. Strong positive effects have been obtained from digital mental health programs for anxiety and depression (eg, g=0.80 and ds=0.49-1.14) [11,12] and small-to-moderate effects for alcohol use [13,14]. Although having a therapist or coach to guide the use of these interventions assists in maintaining engagement and appears to give somewhat better outcomes [15], significant effects can also be obtained by self-guided programs [11,12,16], and their low unit cost means that self-guided programs are easier to take to scale and have superior cost-effectiveness as numbers increase [17]. In some direct comparisons, coached and self-guided programs have even been able to achieve similar treatment outcomes [18]. Although self-guided programs achieve these effects without a therapeutic relationship, we argue that they potentially satisfy other elements of a therapeutic alliance such as perceived safety and consistency with personal goals and avoid many negative effects of face-to-face therapy, such as perceived judgment or stigma. Interestingly, the scores on therapeutic alliance measures from users of self-guided programs can be quite high [19]. It is plausible that a social robot (a robot that can communicate and interact with people) could offer education, model some skills, and deliver a fixed intervention program. As we discuss later in the paper, even more sophisticated therapy may be offered in the future, with emerging developments in robotic technology.

### Existing Reviews

Identified systematic reviews are for social robot interventions in highly specialized areas of elderly care [20,21] and autism spectrum disorder (ASD) [22,23]. They contain a mixture of experimental methodologies such as single subject, quasi-experimental, cross-sectional without control, and free interaction. Mixed-trial designs have generally been considered acceptable when evaluating the initial prospect of a novel intervention [24,25]. However, robotic interventions do require critical evaluation using a series of high-quality trial designs to demonstrate sufficient evidence to achieve effective health outcomes. Current reviews that contain mixed-experiment designs present a limitation around the conclusive nature of identified experimental studies, especially when appraising the use of robotic interventions in routine clinical practice. A high-impact method of clinical trial design involves a randomized controlled trial (RCT), which reduces the influence of bias and confounds on trial outcomes by scientifically rigorous methods of intervention testing to assess treatment benefits [26]. Several systematic reviews using RCTs in surgical robotic interventions have been published [27,28], but evidence has yet to be synthesized for RCTs for social robots to deliver psychological interventions for health or well-being across all age groups.

### Aim

The aim of this systematic review was to undertake a comprehensive examination of existing RCTs on the use of social robots to deliver psychosocial interventions for health or well-being. The review is timely, given the fast pace of developments in robotics, the rapid uptake of social robots that is likely to occur, and the wide-ranging nature of their potential applications to improved health care and self-management support.

## Methods

### Literature Review and Selection of Trials

This systematic review protocol followed the Preferred Reporting Items for Systematic Reviews and Meta-Analyses guidelines for its search, screening, and evaluation processes [29]. Database searches were conducted in Medline, PsycInfo, ScienceDirect, Scopus, and Engineering Village in November 2018. Health, psychology, engineering, and computer science databases were chosen to maximize the chance of identifying published trials that fulfilled the search criteria. Each search used (Title: Robot*) AND (Abstract: Health* OR Anxi* OR Depress* OR Distress* OR Disorder* OR Autis* OR Dement*) AND (Abstract: Therap* OR Behav* OR Treat* OR Intervention* OR Counsel* OR Psychosocial OR Psychotherap*) AND (Title:Abstract:Key: Random*). Medline and PsycInfo searches used Boolean and phrase search modes and included all the results for source types and years. ScienceDirect and Scopus searches were refined to include all years with no document type exclusions in the result searches. Engineering Village contained the GeoRef, Inspec, and Compendex databases, including all years and no document type exclusions. The identified papers from the databases were supplemented by backward and forward searches (ie, checking titles in reference lists and citations of identified papers for any additional studies).

### Inclusion and Exclusion

The eligible trials for the review (1) used a social robot to deliver a psychosocial intervention for health or well-being (ie, one that used verbal communication or other social interaction) and (2) examined the effects of at least 2 conditions in an RCT over at least 2 measurement occasions. A social robot was defined as a humanoid or nonhumanoid robot that could communicate or interact with people using verbal or nonverbal communication or both. These robots could vary from ones with rudimentary abilities (eg, minor motor movements and no communicative speech) to ones with advanced communicative abilities designed to present the illusion of social intelligence. The included trials could use robots operated by staff using Wizard of Oz controls, given that the outcomes of robot-delivered interventions were of greater interest than the ability of robots to deliver interventions autonomously. However, trials using technological agents without embodiment (eg, chatbots or avatars) were excluded, as were ones using robotic devices without communicative abilities, such as prosthetic devices and teleoperated, surgical, and exoskeletal robots.

The papers could have been published in any year. In recognition of the acceptability of conference proceedings as publication outlets for engineering and computer science, the papers could be in a peer-reviewed journal or conference proceedings. Multiple papers on different aspects of a single trial were all used to provide information, but if multiple papers presented the same material, the most complete and current report was selected for evaluation and review.

### Data Extraction and Analysis

Data extraction was conducted in November 2018 by NLR and TVC and reviewed for consistency and accuracy by all 3 authors. All eligible papers were extracted directly from academic databases. The authors were not contacted to provide additional data or unpublished results. Trial extraction involved an initial screen, assessing titles for relevance. Selected papers were further appraised using abstracts, and papers that appeared to meet the criteria were independently reviewed for eligibility and coding in the presented tables by NLR and DJK. In cases of any disagreement in inclusion or coding, the point was checked in the paper and consensus was reached on the final decision. Human-robot interaction factors such as acceptability, likability, and trust of the robot were not reviewed in detail as that lay outside this review. The presented results in the tables include statistically significant ones only: other listed measures were not used as outcomes or did not give significant results.

## Results

**Figure 1:**
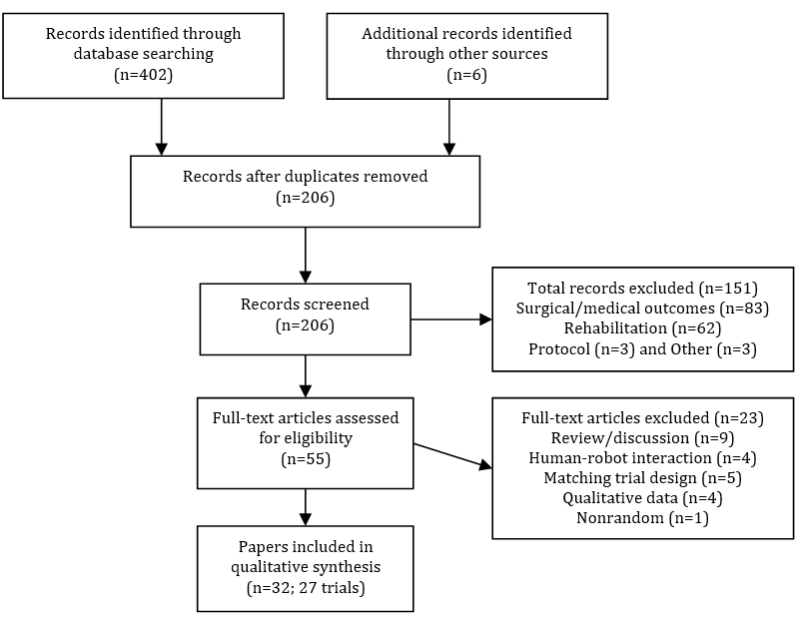
Systematic review flow chart.

The initial search identified 402 records from 5 databases, plus 6 that were identified through forward and backward searches. Identification of duplicates using title, year, and authors resulted in 202 records being removed, leaving 206 for screening. A total of 151 were excluded based on irrelevant titles (eg, surgical and medical trials, exoskeletons, protocols, and economic analyses) and 23 were excluded based on a detailed examination of the abstract and full text, leaving a total of 27 trials for full evaluation. The details of reasons for exclusion are in Figure 1.

### Included Trials

Overall, 6 of the 27 included trials (22%) addressed child health or well-being, 9 (33%) were on children with ASD, and 12 (45%) were on older adults and focused on cognitive or psychological functioning. The most commonly used robots were the NAO humanoid from Softbank Robotics [30], and the PARO harp seal companion robot from PARO Robots [31] (7 trials), although 13 other robots were each used in at least 1 trial. Owing to the wide range of measures and the limited consistency in the presentation of results in different trials, it was not possible to conduct a meta-analysis or to report effect sizes in a standard manner. Accordingly, the systematic review is descriptive.

### Child Interventions for Health or Well-Being

The 6 trials on children’s health or well-being are summarized in Table 1. All had the individual child as the unit of randomization, but only Beran et al [32] and Jibb et al [33] reported computerized or Web-based randomization. Sample sizes ranged from 5 to 57 (median=34). Participants were aged from 4 to 14 years (median reported average=9.9 years), and samples were drawn from Canada, the Netherlands, and Iran. Durations of studies ranged from 1 to 18 weeks (median=4.5 weeks), and no trials had a follow-up assessment. Only Jibb et al [33] reported blind observational coding. All used NAO robots, with preprogramming [32,33] or Wizard-of-Oz individualization [34,35]. The number of treatment sessions ranged from 1 to 10 (median=3).

One study [35] demonstrated a significantly greater rise in diabetes knowledge when a social robot administered a diabetes quiz to children with type 1 diabetes compared with a usual care control. A more personal robot elicited greater pleasure and feelings of self-determination from the participants during the final session, but there were no differences between the robot types on diabetic knowledge. In addition, 3 studies obtained reductions in negative emotions when a social robot was used to assist needle insertion or to address emotional responses in oncology patients or children learning a foreign language [32,36,37]. Less pain was reported about needle insertion in the study by Beran et al [32] and less avoidance to the needle insertion in both the studies by Beran et al [32] and Jibb et al [33]. None of the studies assessed sustained changes in distress, quality of life, health-related behavior, or health outcomes. Although this set of studies provided some evidence in favor of robot use in children’s well-being, conclusions were limited by infrequent blind assessment and a lack of follow-up data or information on behavioral or functional impacts. Research in this area is at a very early stage.

### Children Interventions for Autism Spectrum Disorder

Overall, 9 trials of robot interventions for children aged 4 to 12 years with ASD or pervasive developmental disorder were identified (Table 2). In addition, 6 trials randomized individuals to conditions, 2 randomized to condition order, and 1 had cluster randomization. None of the trials reported independent randomization, and only 2 trials [38-41] reported using computer-generated randomization. Moreover, 3 studies [40,42,43] had a single-blind assessment, and all but 1 study [44] reported that the reliability of observations was confirmed against another rater (in that study, a single rater was used, but reliability was established in training).

**Table 1:** Child interventions delivered via a robot for health or well-being.

Author	Sample	Design, conditions (n)^a^	Duration	Measures	Outcomes^b^
Beran et al [32]	57 Canadian vaccination patients (30 male, 53%), aged 4-9 years (mean 6.9, SD 1.3)	Robot CBT (28): Distraction before, during, and after injection; control (29): standard nurse administration	1 session^c^ at vaccination	Faces Pain Scale-Revised (FPS-R); Behavioral Approach—Avoidance Distress Scale (BAADS)	Robot Cognitive-Behavior Therapy versus control during session: <pain (FPS-R) from parent, child*, nurse*, and researcher and* <BAADS Distress**, Avoidance***
Blanson Henkemans et al [34]	5 Dutch type 1 diabetes patients (3 male, 60%) aged 9-12 years (mean 10.2, SD 1.3)	Game-like quizzes (10 out of 20 of the questions on diabetes each session); personal robot (3): eyes in favorite color, used child’s name, mentioned child’s favorite activity, asked opinion of game, if wanted to keep playing, etc; and neutral robot (2): no personalization	3 sessions (45, 45, and 30 min) at 2-3 week intervals	Type 1 diabetes knowledge; health-related quality of life; and Mind Youth Questionnaire (MY-Q)	Across conditions, Pre to Session 3^d^: > correct diabetes questions*
Alemi and Meghdari [36]	46 Iranian female students aged 12-13 years, with beginners’ level English	Individual randomization to classes; robot-assisted language learning (RALL; 30 in 2 groups); and control: teacher only (1 group of 16)	10 × 1-hour sessions over 5 weeks	Foreign Language Classroom Anxiety Scale (FLCAS); attitude questionnaire	RALL versus control at 5 weeks: >FLCAS (less anxiety)*
Alemi et al [37]	11 Iranian oncology patients (1 male, 9%) aged 7-12 years (mean 9.5, SD 1.6)	Social robot-assisted therapy (SRAT, 6): robot took roles of doctor, chemo-hero, nurse, cook, ill kid; shared hopes and dreams, said goodbye and control: psychologist only (same content; 5)	8 sessions^c^ over 1 month	Multidimensional Anxiety Children Scale (MASC); Children’s Depression Inventory (CDI); and Children’s Inventory of Anger (CIA)	SRAT versus control, pre versus 1 month: >falls in anxiety (MASC)**, depression (CDI)*, and anger (CIA)*
Blanson Henkemans et al [35]	28 Dutch type 1 diabetes patients (13 male, 46%) aged 7-14 years (mean 11.0, SD=1.7)	Diabetes education quizzes; Personal robot (9): as per Blanson Henkemans et al [34]; neutral robot (8): no personalization; and control (11): no robot or quiz	Robot groups: 3 sessions (50, 40, and 40 min), 6 weeks apart	Diabetes knowledge; quiz rounds decided to play, desire to play in a fourth session, rated pleasure; behavior during interaction; and Basic Need Satisfaction in Relationships Scale	Combined robot groups versus control^e, f^: >correct diabetes knowledge questions after Session 3^;^*** personal versus neutral robot: >quiz rounds in Session 3; > number electing to play a fourth session; > on some positive behaviors during some sessions (eg, smiling at the robot in all sessions); > perceived self-determination on BSNR* during Session 3
Jibb et al [33]	40 Canadian cancer patients (24 male, 60%) aged 4-9 years (mean 6.2, SD 1.5)	Cognitive-behavioral robot (“MEDiPORT”, 19): supportive statements, deep breathing exercises; active distraction Robot (21): Introduction statement, dancing moves while singing	1 session at subcutaneous needle insertion appointment	BAADS; Face Pain—Revised (FPS-R); Children's Fear Scale; and Acceptability questionnaire (Likert and free text)	Active distraction robot: < avoidance during nurse movement toward child**, at needle insertion^;^** and < parent-rated acceptability of time to conduct needle insertion*

^a^All studies used the NAO robot, and all were randomized controlled trials with individual randomization. Numbers are at allocation.

^b^Effects on measures not reported under results were not statistically significant.

^c^Duration was not reported.

^d^Differences between effects of the 2 conditions were only reported descriptively.

^e^Results reported on 27 patients (1 neutral robot participant dropped out before session 1).

^f^Personal versus neutral robot effect for knowledge not reported.

*P<.05.

**P<.01.

*** *P*≤.001.

**Table 2:** Child interventions delivered via robot for autism spectrum disorder.

Author	Sample	Design, conditions (n)^a^	Duration	Measures	Outcomes^b^
Kim et al [45]	24 US children with autism-spectrum disorder (ASD); 21 male, 88%) aged 4-12 years (mean 9.4, SD 2.4); Autism Diagnostic Observation Schedule (ADOS): 20 met criteria for autism, and 4 for autism spectrum disorder	Random order within subject: Pleo robot interaction, adult interaction and computer game	1 session: 3 × 6-min interactions, each separated by 6 min of interview and play	Verbalization (number of utterances produced)	Robot segment: > total speech versus adult*, Computer game***; >speech to confederate versus Adult*, computer game***; > speech to Pleo than computer game***, Pleo versus Adult not significant (ns)
Huskens et al [46]^c^	6 Dutch males with ASD aged 8-12 years (mean 10.50, SD 1.37); all had Social Communication Questionnaire (SCQ) >15 (range 18-32)	Random order within subject: robot: NAO; human trainer; robot and human made statements inviting a question and performed requested actions (eg, dance)	Introduction to robot (2 sessions); baseline—4 robot, 4 human 10-mi training sessions; and follow-up 2 weeks after last training	Question-asking (number of self-initiated questions) in 3-5 × 10-min sessions with human assessor at baseline and follow-up	Both conditions, baseline session 1 versus intervention and follow-up^d^: >correct questions during training, maintained at follow-up
Pop et al [47]	20 Romanian children (sex not stated) with ASD aged 4-9 years; no significant between-group differences on Children’s Autism Rating Scale (CARS)	Randomization in clusters of 3: story telling; Probo robot-assisted therapy social stories (SS-RAT, 7); computer-presented social stories (SS-PC, 6); and control (7)	SS-RAT and SS-PC: 6 sessions^e^; control: 4 × 10-min observations on different days	Social expression (degree of prompt required for social response)	SS-RAT versus control at post^e^: > social expression*; (SS-PC versus control ns)
Peca, Simut [48]	27 Romanian children^f^ (22 male, 82%), 18 with ASD, 9 with pervasive developmental disorder (PDD), aged 4.5-8 years (mean 6.2, SD 1.0). No significant between-group difference in mean ADOS (Robonova: 15.00; adult: 15.09)	Contingent (imitating child) and noncontingent play, with: Robonova robot (12) and adult (9)	1 session: 2 × 80-second segments^g^ separated by a 5-min pause	Social intention (eye gaze, positive affect, initiations, intention testing, tests per initiation frequency); contingent (mirrored behavior)	Robot versus adult: > eye gaze*** (contingency ns)
Srinivasan et al [38,39,41,49]	36 US children with ASD (32 male, 89%) aged 5-12 years (mean 7.6, SD 2.2) ADOS-2 range 6-10 (means—Robio: 8.5, rhythm: 7.9, and control: 8.4)	Robot (12): NAO and Rovio, whole-body imitation and interpersonal synchrony games; rhythm (12): human, singing and whole-body imitation games; and control (SC, 12): tabletop activities (academic, communication, and fine motor)	32 sessions over 8 weeks (post at 10 weeks)	Joint Attention Test (JTAT); social verbalization; imitation, praxis, interpersonal synchrony; Bruinicks-Oseretsky Test of Motor Proficiency (BOT); Repetitive and maladaptive behaviors; and Affective states	Robot versus control^h^: >attention to human partner, elsewhere***^i^; <attention to objects***; > spontaneous human attention***; > self-directed vocalization**; *< human social vocalization* ***; < spontaneous human social vocalization*; < sensory behaviors in late session**; > negative affect*; < interested affect*; and < fine motor control at Post*. Robot versus Rhythm, during session^h^: *<attention to human partners and elsewhere* ***^i^; *< spontaneous human attention* ***; > self-directed vocalization**; < spontaneous human social vocalization*; and < positive affect in mid & late sessions**. Group x Early, Mid, Late Session: Words in response to questions*** (only Rhythm rose). Robot, pre and post: > body co-ordination* and > imitation**. Robot, early versus late session: <positive affect* and > time in-synchrony*
Costescu et al [44]	27 Romanian children with ASD (20 male, 74%) aged 6-12 years (mean 8.7, SD 1.8); ADOS-Generic (mean 10.32)	Robot-enhanced therapy (14, RET)^j^: My Keepon, distinguishing emotions from 15 social situations; discussion: cognitions, emotions and behavior connections; adaptive strategies for anger, self-control and control (n=15, standard care [SC])	RET: 6 × 2-hour weekly group sessions	Frequencies of correct strategies in a social situation; rational or irrational beliefs; adaptive behaviors; and emotional intensity	RET versus control, post (controlling pre): >rational beliefs** and <(negative) emotion intensity***
Yun et al [40]	15 Korean males with ASD aged 4-7 years (mean 5.8, SD 0.9). No significant between-group differences on ADOS subscales or current SCQ (lifetime SCQ higher** and IQ lower* in robot group)	Social skills training (eye contact and reading emotions). Robot (8): iRobiQ (4 weeks), CARO (4 weeks) and human trainer (7)	8 × weekly 30-40 min sessions (post at week 9)	Autism Diagnostic Observation Schedule (ADOS, by blind rater); Vineland Adaptive Behavior Scale (Korean version); Social Communication Questionnaire; Social Responsiveness Scale; and Child Behavior Checklist (Korean version, CBCL)	No differences robot, human; both (versus pre): < (better) ADOS Play*; <CBCL Internalizing at post* (Depression and Anxiety*, Withdrawal* subscales); >frequency of eye contact, Session 8*; >recognition accuracy of most difficult facial emotions by Session 4*
So et al [42]	13 Hong Kong children (10 males, 77%) with ASD aged 6-12 (mean 9.0, SD 2.4) ADOS scores not reported (nr)	NAO Robot (7); control (6): educational videos; for both, phase I: Recognize 8 gestures; phase 2: Produce 8 gestures	In each 6-week phase: 4 × 30-min sessions over 2 weeks; tests pre, post, and 2 -week follow-up	Phase 1: Recognize gestures; phase 2: Produce gestures; tested on 2 trained gestures, 2 untrained; 20% of ratings rescored by a blind rater	Phase 1 recognition, robot versus control: pre and post: >scores on trained***, generalized***, human-to-human*** gestures; post follow-up: ns; phase 1 production, robot versus control: pre and post: >scores on trained**, generalized^k^, human-to-human ns; post follow-up: ns
So et al [43]	45 Hong Kong (Cantonese-speaking) children (36 males, 80%), aged 4-6 years; 30 with ASD (3 female): intervention (mean 5.8, SD 0.8) waitlist (mean 5.7, SD 0.4); 15 age-matched controls (6 female) (mean 5.3, SD 0.7); and ASD severity nr	NAO robot demonstrates and elicits gestures while narrating stories; intervention (15); waitlist (15); age-matched, no ASD control (15)	Over 9 weeks: 4 × 30-min training sessions for 14 gestures (2 sessions per week); tests at pre, post, 2-week follow-up (2 test sessions each)	Gestural production in training, novel stories (10 seconds to respond, prompt and another 10 seconds if no response); gestural recognition; psychoeducational—third edition; Bruininks-Oseretsky Test of Motor Proficiency 2nd Edition (BOT); and Attention Network Task (ANT)	Gestural production (pre, post, follow-up), controlling for language and developmental age, BOT, ANT, gestural recognition^k^: Group × Time***, Group × Training and Novel ***, Group × Time ×Training and Novel*** control > Intervention*, Wait List* at Pretrained: intervention > waitlist (post***, follow-up***); > control (Post***, Follow-up*); -Novel: Intervention > Wait List (Post***, Follow-up**); = Age-matched controls; Intervention versus Wait List (Pre to Follow-up) with covariates as above: Verbal imitation: Group × Time* (only Intervention group increasing*)

^a^Randomized controlled trial with the individual participant as the unit of randomisation unless labeled otherwise.

^b^Effects on measures not reported under results were not statistically significant. Some results that did not involve the robot condition are omitted. Results where the robot did significantly worse than the comparison condition are italicized.

^c^Differences between effects of the 2 conditions were only reported descriptively.

^d^Analyses of changes within conditions are reported separately, as are effects for each individual.

^e^Total period of training and timing of post not reported.

^f^An additional 6 children were excluded because they refused to undertake the tasks.

^g^The paper refers to the session segments as sessions.

^h^ Results from these studies were incompletely reported, and some reporting is ambiguous. Effects are across sessions unless otherwise stated.

^i^The attention target analysis appears inappropriate (only the robot group could have attention to the robot, affecting analysis of condition effects). “Elsewhere” is attention other than to the human partner, robot, or objects.

^j^Analyses were on 12 RET (2 withdrew); 15 control participants.

^k^Recoding for gestural appropriateness rather than strict accuracy was interpreted as supporting these results, but only gave Group effects (using pre and follow-up only).

*P<.05.

**P<.01.

***P≤.001.

Sample sizes ranged from 6 to 45 (median=24). However, only 3 recent papers reported some blind coding of observations [40,42,43]. The studies had 1 to 32 sessions (median=4), and study durations ranged from 0 to 14 weeks (median=9 weeks, 2 studies were unclear). In addition, 4 studies [38,41-43,46] reported a follow-up, all were of only 2 weeks. All but the small study by Huskens et al [46] presented the results against a comparison condition: they reported the results within each condition and within each participant.

Participants were aged from 4 to 12 years (median reported mean age=8.7 years). Samples were drawn from the United States (2 studies), Romania (3 studies), Hong Kong (2 studies), and the Netherlands and Korea (1 study each). The nature and roles of the robot were also diverse. Some studies used the robot as an assistive tool to therapist interventions [47], whereas in others, it was the primary method of therapy delivery [40]. All but 3 studies (78%) had a researcher who was operating the robot using Wizard-of-Oz control. The remaining studies used a set program where the researcher could pause the program if needed [42,43] or where limited branching was produced by eye contact or by the researcher pressing a button to record the child’s response [40]. The number of treatment sessions ranged from 1 to 32 (median=4) over a period of 1 to 8 weeks (median=2 weeks, 2 unknown). Despite the small sample sizes in these trials, positive effects were found on several measures; although inspection of Table 2 shows that differential results on many measures were not statistically significant. In relation to changing beliefs, a robot to deliver therapy increased the presence of more rational beliefs [44]. For improving emotional affect, the identified trials resulted in decreased negative emotion intensity [44] and lower scores on depression, anxiety, and withdrawal subscales after treatment [40]. Social behavior improvements were present with increased eye contact [40], gaze frequency in the direction of the interaction partner [48], increased levels of social expression [47], higher total number of produced verbal utterances [45], recognition accuracy of facial emotions [40], and number of correct questions [46], as well as improved gestural recognition [42] and production [43]. Robots could achieve greater effects than a standard care control [44,47], educational videos [42], and a computer game [45].

Effects of the robot versus a human trainer were typically the same [40] or superior on at least 1 measure [48]. The 1 exception was a study by Srinavasan et al [38,39], where average effects of the robot were never superior to the human, and the human condition did better on several social indices. However, that study confounded the actor (human versus robot) and intervention content (eg, the human condition used singing and the robot one did not). Furthermore, calculation of the focus of attention was affected by the fact that a focus on the robot was not counted as attention to the social partner, and the superior result on fine motor skills in the control group may be ascribed to a difference in fine versus gross motor tasks in the 2 conditions rather than to use a robot per se. The observation of less interested affect and more negative affect in the robot condition than in controls deserves further attention, although it appears inconsistent with positive effects on negative moods that were seen in the studies by Costescu et al [44] and Yun et al [40]. Overall, the results by Srinavasan et al appear at odds with those from other trials and are subject to methodological limitations.

In summary, the strengths of these trials included their substantiation of interobserver reliability and the fact that a third had some blind assessment, and 4 trials had a follow-up assessment (albeit only 2 weeks later). Social robots for young people with ASD appear to have positive outcomes, although studies with larger samples and longer follow-ups are needed to build confidence in the strength and sustained maintenance of these effects.

### Interventions for Older Adults

Overall, 12 trials of robot interventions for older adults were identified (Table 3). Most aimed to improve cognitive and or psychological functioning or neural integrity, although 1 focused on self-management of chronic obstructive pulmonary disease (COPD) [50]. Where mean ages of participants were reported, they ranged from 67.4 to 85.3 years (median=84 years). In addition, 4 studies were from New Zealand, 2 from Australia, 2 from the United States, and 1 study from Korea, Norway, Spain, and Japan each. Moreover, 8 trials were conducted in residential facilities, but 4 used an intervention in the home or day care center. Furthermore, 8 trials randomized individuals (2 of these to a random order of conditions); 4 had cluster randomization (1 to a random order).

**Table 3:** Adult interventions delivered via robot.

Author	Sample	Design, Conditions (n)^a^	Duration	Measures	Outcomes^b^
Banks et al [51]	40 US residents of long-term care facilities scoring ≥24 on the Mini-Mental State Examination (MMSE) and ≥30 on University of California, Los Angeles (UCLA) Loneliness Scale (age and gender nr)	Interactions with robot (15)^c^-AIBO robot dog or living dog (15); control (13)—no intervention	Robot, dog: 8 weekly 30-min sessions	UCLA Loneliness scale; Lexington Attachment to Pets Scale	Robot or dog versus control, pre and post: >fall in loneliness* (robot=dog)
Tanaka et al [52]^d^	34 female Japan residents aged 66-84 years	Kabochan Nodding Communication ROBOT (18): Communicate by talking and nodding and control (16): same robot but no talking or nodding	Robot at home for 8 weeks	MMSE; Cognistat test; Blood and saliva samples; Accelerated plethysmography; Questionnaire: Appetite (visual analogue scale) sleep; depressive symptoms (Geriatric Depression Scale [GDS-15]); Activities of daily living—Tokyo Metropolitan Institute of Gerontology Index of Competence	Communication Robot: > MMSE score after 8 weeks**; > Verbal memory after 8 weeks*; > Everyday/concrete judgements after 8 weeks*; > Attenuation of fatigue compared with control*; > Enhancement of motivation compared with control**; and >healing compared with control*
Robinson et al [53]	40 New Zealand retirement home residents (13 men, 33%) aged 55-100 years	Robot (20)^e^: PARO—interactions with robot and control (20): alternate activities)	Robot: 2 group sessions per week for 3 months	UCLA Loneliness scale; GDS-15; and Quality of Life for Alzheimer’s Disease (QoL-AD)	Robot versus control, pre and post: >fall in loneliness*
Moyle et al [54]	18 Australian residential aged care residents (sex not stated) aged ≥ 65 (mean 85.3, SD 8.4)	Within-participant crossover design (random order); Robot first (1 group of 9): PARO -discovery, emotional response, discussion about PARO, touching PARO and control first (9): Being read to, looking at pictures, discussion of readings	Each phase: 3 × 45-min 9-member sessions per week over 5 weeks; 3-week washout between phases	Quality of Life for Alzheimer’s Disease Scale; Rating Anxiety in Dementia Scale (RAID—self-reported and Proxy); Apathy Evaluation Scale; Geriatric Depression Scale; Revised Algase Wandering Scale Nursing Home version; Observed Emotion Rating Scale (OERS); (Assessors independent—unclear if blind)	Robot versus control after intervention (reporting range of Cohen *d*)^f^: >Quality of Life (0.6 to 1.3); < anxiety on RAID Proxy version (−0.4 to −0.3) but greater on RAID (0.4 to 0.4), OERS (0.5 to 0.7)^f^; > OERS sadness (0.4 to 0.6), pleasure (0.7 to 0.7)^f^
Broadbent et al [55]	29 New Zealand retirement village residents (14 male, 48%) aged 72-94 years (mean 85.2, SD 5.1)	Within-participant crossover design (random order)^g^; iRobiQ or Cafero robot at home versus control—measured blood pressure and pulse oximetry, had music and quotes; iRobiQ: also medication reminders, alert to nurse if not taken or said unwell; and Cafero: cognitive exercises, village map, and calendar reminder	2 × 6-week periods with 18-day washout	Geriatric Depression Scale; Health-related Quality of Life; and Medication Adherence Report Scale (Single-blind assessment)	iRobiQ or Cafero versus control pre and post: not significant (ns)
Kim et al [56]	85 Korean community residents (25 male, 29%) aged > 60 (mean 67.4) with MMSE Korean version > 26 (mean 29)	All: 10 hour dementia prevention education on before baseline; cognitive training: robot (24)^h^: Silbot and Mero -17 training programs with individual rewards immediately after smart pad answers; winner of day, month; traditional cognitive training (24)^h^: question and answer display; nonrandom control (37), no training)	Education: 2 hours per day over a week; cognitive training: 60 × 90-min 8 -member sessions over 12 weeks	MRI cortical thickness, intracerebral volume, structural connectivity; Alzheimer’s Disease Assessment Scale-Cognitive Subscale (ADAS-Cog); Cambridge Neuropsychological Test Automated Battery; Delayed Matching to Sample; Pattern Recognition Memory (PRM); Paired Associates Learning; Spatial Working Memory; Stockings of Cambridge (SOC); Reaction Time; Rapid Visual Information Processing (Blind scoring of all assessments)	Cognitive training versus control, pre and post: <reduction in cortical thickness*, nodal strength*, global efficiency*, clustering coefficient* > executive function (SOC)*** (robot=traditional); robot versus traditional: <cortical thinning in right and left anterior cingulate, areas of right inferior temporal cortices***; > nodal strength, left rectus gyrus***; and < improved on ADAS-Cog* and PRM*
Valenti Soler et al [57]	Spanish nursing home patients with dementia; phase 1: 101 adults (12 male, 11.8%) aged 58-100 (mean 84.7); phase 2: 110 adults (11 male, 10.0%) aged 59-101 (mean 84.7)	Cluster randomization by living unit; all: training—for example, identifying numbers, words, colors; use of everyday objects; sensory stimulation; phase 1: assisted by PARO (33), NAO (30), and control (38); 9-month washout; phase 2^i^: Assisted by PARO (42), dog (36), control (32); Day Care (Nonrandom); phase 1: assisted by NAO (20); 9-month washout; and phase 2^i^: assisted by PARO (17)	30-40-min group or individual sessions × 2 days per week × 3 months	Global Deterioration Scale; Severe Mini Mental State Examination; Mini Mental State Examination (MMSE); Neuropsychiatric Inventory (NPI); Quality of Life in Late-stage Dementia (QUALID); Apathy Scale for Institutionalized Patients with Dementia Nursing (Home version; APADEM); Apathy Inventory (single-blind assessments)	Nursing home phase 1; NAO versus control, pre and post: > reduction in APADEM total*, Cognitive inertia subscale*: > reduction in NPI apathy/indifference*; worse delusions*; < (worse) mental state (MMSE)*; PARO versus control, pre-post: > reduction in APADEM total*; > NPI Irritability/lability* phase 2 PARO versus control: > quality of life (QUALID)*; > NPI hallucinations*, irritability/lability** Day Care phase 1 (NAO): < NPI total**, Irritability/lability* phase 2 (PARO): ns
Jøranson et al [58,59]	60 Norwegian nursing home patients (10 male, 33%), aged 62-95 (mean 84) with dementia or MMSE < 25	Cluster randomization of 10 living units; robot (30)^j^: PARO; control (30)^j^: SC	Robot: 2 × 30-min sessions (≤6 members) per week for 12 weeks; tested at pre, post, 3-month follow-up	Brief Agitation Rating Scale (BARS, interrater reliability reported); Cornell Scale for Symptoms of Depression in Dementia (Norwegian, CSDD); Medication; QUALID	Robot versus control, Pre and Follow-up^j^: < agitation (BARS)*; < depression (CSDD)*; > quality of life (QUALID, severe dementia patients only)*; robot versus control, Pre and Post: <medication, severe dementia patients only*
Liang et al [60]	30 New Zealand dyads: patients with dementia (11 male, 36%), aged 67-98; caregivers (4 male, 13%), aged 30-86)	Robot (15)^k^: PARO, at day care and at home and control (15)^k^: SC	Robot, over 6 weeks: 2-3 × 30 -min sessions per week (day care, groups of 3 -6) and ad lib at home; tested at pre, 6, 12 weeks	Behavioral, affective, and social responses during sessions; Blood pressure; salivary cortisol; Addenbrookes Cognitive Examination (NZ version); CSDD; Neuropsychiatric Inventory Brief Questionnaire Form; Cohen-Mansfield Agitation Inventory (Short Form); and Hair cortisol	Robot versus control, Pre, 6 and 12 weeks: > drop in depressive symptoms (CSDD) but increase between 6 to 12 weeks (interaction effect*). Robot versus control, during sessions: >Happy, smiling facial expressions (Agitation, social interactions ns)
Petersen et al [61]	61 US patients in assisted living memory care units with mild-moderate dementia (14 male, 23%) aged ≥ 60 (mean 83.4)	Cluster randomization by coin toss: Robot (35): PARO and control (26): SC activities	Both: 3 × 20-min sessions per week (6 members) for 12 weeks	Global Deterioration Scale (interrater reliability reported) RAID; CSDD; Galvanic skin response (GSR); pulse rate; pulse oximetry; and medication doses	Robot versus control, pre and post: > rise?^l^ in anxiety (RAID)**, depression (CSDD)***; > rise?^l^ in GSR***, pulse oximetry***; > fall in pulse rate***; and >fall in doses of pain medication*** and behavior medication***
Broadbent et al [50]	60 New Zealand patients (aged between 16-90 (mean 69.8, 62% female) with chronic obstructive pulmonary disease (COPD), recruited at inpatient discharge	Robot (30)^m^, iRobi at home: weekly clinical assessments; reminders to take medication, inhalers, do rehab exercises; education in videos, pop-up messages; “I am feeling unwell” button (initiating clinical assessment, message to staff); display trends in status, adherence. Linked to SmartInhaler alert to staff if missed medications, exercise 3 times. Phone calls to follow-up alerts, remind to use robot and control (30)^m^, SC	4-month robot use	Quality of life—Clinical COPD Questionnaire; medication adherence—Medication Adherence Report Scale—and Frequency of rehabilitation exercise	Robot versus control, pre and post (controlling for comorbidities, past hospitalizations): hospitalizations (primary outcome) ns; > self-reported medication adherence* (electronic inhaler only before covariates); > self-reported rehab exercises***; robot versus control: <direct cost (saving NZ$1152; *d*=.27), total hospitalization cost (saving NZ$1579; *d*=.27)
Moyle et al [62] and Jones et al [63]	415^n^ Australian residential patients with dementia (100 male, 24.1%) aged >60 years (mean 84-86 in each condition)	Cluster randomization (N facilities, participants); PARO (9, 138)^n^; Plush toy—PARO with robot features disabled (10, 140)^n^; SC (9, 137)^n^	PARO and Plush Toy: 3 × 15-min individual, non-facilitated sessions for 10 weeks (ie, 30 total) and assessed at pre and weeks 1, 10, 15 (post)	Positive behavioral engagement, mood states and agitation (video observation); Cohen-Mansfield Agitation Inventory-Short Form; Rowland Universal Dementia Assessment Scale; Multicultural Cognitive Assessment Scale; Using SenseWear Professional 8.0 activity armband: Day and nighttime motor activity (steps, hours of physical activity) and hours lying down, asleep, and awake	PARO versus Plush Toy, pre and post^o^: > verbal* (.011), visual engagement***; < steps in day*, nightime*^p^; < hours physical activity*^p^. PARO versus SC, pre and post^o^: > neutral* and pleasure** affect; < agitation**; < steps in day*^p^. PARO and Plush Toy versus SC, pre and post^o^: > neutral affect**

^a^Randomized controlled trial with the individual participant as the unit of randomization unless labeled otherwise. Numbers are at allocation.

^b^Effects on measures not reported under results were not statistically significant. Some results that did not involve the robot condition are omitted. Results where the robot did significantly worse than the comparison condition are italicized.

^c^Analyzed 13 robot, 13 dog participants.

^d^Random assignment matched for age and MMSE score.

^e^Analyzed 17 robot (3 died), 17 control participants (2 died, 1 moved away).

^f^Text says the amount of missing data was large, and no substitution for missing data was made. However, tables give an n of 18. Analyzed by standardized mean difference between scores after each intervention. Results with Cohen *d* ≥0.3 are displayed (range in brackets).

^g^Number in each order not reported. Individual randomization, but mentions 2 participants who were married and living together.

^h^Excluded 2 robot, 1 traditional participant from MRI analyses (similarity index <0.5).

^i^Some overlap of phase 2 participants from phase 1. Loss to analyses: nursing home phase 2 dog (1); day care phase 1 (2), phase 2 (2).

^j^Lost 2 robot, 4 control participants who died; 1 robot participant withdrew. However, analyses used intention to treat (by imputation, mixed models).

^k^Analyses on 13 PARO, 11 SC participants.

^l^All of the results are described in the text as greater improvements in the robot condition, but mean changes presented in Table 2 on the RAID, CSDD, and GSR show larger positive post minus pre changes in the robot condition. That would indicate greater deterioration. A question mark is used to highlight the issue.

^m^Hospitalizations were reported as intention to treat (omitting 1 who died) and per protocol. Most other results (referred to as *intention to treat*): were on 25 robot participants (3 withdrew, 2 died), 26 controls (1 withdrew, 1 did not complete follow-up assessments, 2 died). Electronic inhaler results were on 18 robot, 25 control participants.

^n^All allocated participants were in analyses. Losses to assessment postallocation included PARO: 7 deceased, 1 relocated; Plush toy: 14 deceased, 1 palliative care; SC: 5 deceased, 1 palliative care, and 1 relocated.

^o^Secondary analyses examined effects at weeks 1 and 5.

^p^Interpreted as a positive outcome because of association of physical activity with agitation.

*P<.05.

**P<.01.

***P≤.001.

The use of a robot had an impact on emotions, such as achieving an increase in neutral and pleasure affect [62,64], and happy, smiling facial expressions [60], with a decrease in depressive symptoms [58,59] and loneliness scores [51,53]. Reported increases were found in quality-of-life measures [54,57], but for another sample, this was only present for severe dementia patients [58,59]. Cognitive functioning could also be improved in areas such as reducing cortical thickness and improving executive functioning [56]. The use of a robot helped to reduce agitation [58,62,64] and increase other behaviors, such as verbal memory [52]. Robots also assisted with a fall in pulse rate, doses of pain medication and behavior medication [61], increase in self-reported medication adherence, and rehabilitation exercises, with substantial cost saving [50].

In addition, 6 studies reported using independent or blind randomization [50,54,56,58,60,62,63]—in the case of Broadbent et al [50], controlling for ethnicity and gender. Robinson et al [53] reported using a random list generator but did not state other details. In some other trials, the randomization was basic (eg, Petersen et al [61] used a coin toss and Valenti Soler et al [57], a die) or potentially problematic (eg, Broadbent et al [55] randomly allocated at least 1 couple in a study with participant randomization). Where block randomization was used, only Jøranson et al [58] appeared to have analyzed for cluster effects. Kim et al [56] had an additional nonrandom control group, and Valenti Soler et al [57] also reported on a nonrandom study.

The trial durations ranged from 8 to 24 weeks (median=13 weeks). A total of 3 trials had a follow-up assessment: 2 for 5 to 6 weeks [60,62,63] and 1 for 3 months [58]. In addition, 4 trials had a blind assessment of observations [55-57,62,63], and interrater reliability on at least 1 key measure was reported in 3 trials [59,61-63]. Sample sizes ranged from 18 to 415 (median=50). Moreover, 8 trials reported intention-to-treat results on at least some measures data, and retention was generally high. Some data appeared to conflict with the described results in the study by Petersen et al [61].

Identified trials often involved the PARO robot, which resembles a baby harp seal, and is designed to mimic animal behaviors, but avoids attendant risks of injury or infection. PARO is furry; responds to touch, sound, light, posture, and temperature; and has a diurnal rhythm and some interaction capability [31,65]. As a result, it can be reliably used for responsive interactions without Wizard-of-Oz control. However, similar to animals, it cannot verbally communicate, thus limiting the range of social interactions it can undertake. These trials aimed to elicit behavioral, affective, and social responses to improve mood and cognitive functioning.

Table 3 demonstrates that the robotic interventions typically resulted in better cognitive or neural functioning, reduced distress, or better quality of life, although (as may be expected in dementia) positive cognitive or neural outcomes sometimes involved less decline rather than greater improvement [56]. Overall, 2 sets of results were particularly notable: a lesser reduction in cortical thickness and in global efficiency in the trial by Kim et al [56] and (despite the lack of significant differences in hospitalizations) reduced direct costs from treatment of COPD in the study by Broadbent et al [50]. However, consistent with the trials on ASD, many measures did not show differential changes from the robot intervention.

Even more importantly, as shown in the italicized results in Table 3, some trials found that the robot condition had inferior results on some measures. Examples include inconsistent results on emotions in the study by Moyle et al [54] and on cognitive tests in the study by Kim et al [56], and some negative effects on symptoms including irritability/lability in the study by Valenti Soler et al [57]. Importantly, a set of predictive analyses that was undertaken by Jones et al [63] within the PARO condition of the recent study by Moyle et al [62] and Jones et al [63] showed that more positive and visual engagement with PARO was seen in participants with low levels of agitation at baseline. Mild cognitive impairment predicted greater visual engagement with the robot and more pleasure at week 10. These results suggest that positive effects from PARO may predominantly occur in less severely affected participants. A cost-effectiveness study on the recent study by Mervin et al [66] found that the PARO robot gave a slightly lower incremental cost-effectiveness over usual care in reducing agitation than did the Plush Toy (the PARO with disabled robotic features). However, neither cost was substantial.

### Ineligible Trials

Inspection of excluded trials also offers some important insights into the state of current research on robotics in health care. Medical trials retain dominance with 145 excluded papers (83 papers on surgical, rehabilitative, and exoskeletal applications and 62 on rehabilitation and gait training) and that research shows greater maturity than in other health care domains. Other social robot studies placed greater focus on the acceptability of social robots or evaluation of different robot characteristics, demonstrating that potential applications and intervention elements are still being identified [67]. Excluded papers also included trial protocols [68,69], qualitative studies [70], and reviews [71].

## Discussion

### Principal Findings

In comparison with trials on surgical or other medical applications of robots, trials on improving health, well-being, or psychological interventions using social robots are very few; are limited to the contexts of child health, ASD, and older adults; and, as a group, are relatively unsophisticated. Conversely, the contexts where these robots have been trialed present significant challenges for both treatment and research.

In light of those challenges, it is encouraging that all of the reviewed papers provided some evidence of positive effects from an intervention using a social robot, even though several found that some measures showed no differential effects or favored alternate treatments. Furthermore, some null results may be regarded as a positive finding. For example, the fact that Huskens et al [46] and Yun et al [40] found no difference between results from a human and a robot trainer may presage greater cost-effectiveness, if a human operator was not required in similar future applications. Some effects that appeared negative could be attributed to methodological issues with the trial, such as the confounding of content and delivery in the study by Srinivasan et al [39]. However, some other results were more disquieting, such as increased negative affect and less interested affect than controls in the study by Srinivasan et al [39], increases in sadness and (on 1 measure) anxiety in the study by Moyle et al [54], and some worsening of symptoms in the study by Valenti Soler et al [57]. Whether social robots sometimes trigger distress or other negative emotions in dementia needs further examination, as do the ways any negative effects may be avoided or reduced and whether interventions should primarily target people with less agitation or cognitive impairment. Although some of these results were inconsistent with those from other studies and others may have been because of uncontrolled factors, they require further attention to see if they are replicated and identify factors that are triggering them. For example, some participants with ASD or dementia may find specific social robots or robotic behaviors anxiety provoking or greater effort may be needed to acclimatize them to the novelty of interacting with a robot.

Generalizability of benefits from the robotic intervention outside the context-specific tasks was not tested in most trials. For example, child social skills’ training was not explored beyond the context of the session to investigate its translatable impact on other interactions, such as with adults outside of the research team or other children. Only 6 trials (22%) had any follow-up at all, and among these 6, none had a follow-up assessment beyond 12 weeks (range=2-12 weeks, median=5). Most trials were small (median N=34), with only 2 trials having more than 100 participants. A single-blind assessment of observations was reported in just 8 trials (30%). Overall, more recent trials tended to have superior methodological quality, especially in the older adults’ group of studies. However, all 3 areas had significant thematic limitations.

### Limitations

The limitations of the review include its inability to assess average effect sizes because of the wide range of measures and reporting methods in the identified papers. The restriction of trials to the ones reported in English may have missed some trials, and some other databases or search terms may have identified further trials, although the use of forward and backward searches should have reduced the risk of missed trials. Given a publication bias toward significant results, there may be other trials with less positive outcomes than the ones reported. The review is also limited by its focus on published papers and contacting authors may have clarified some methodological features that were unreported or ambiguous. Large-scale RCTs are often subject to extensive testing and recruitment timeframes, and therefore, eligible trials may still be in operation at the time of publication. In addition, trials are restricted by the limited number of social robots available to them, constraining researchers to shape their interventions to fit onto the current capabilities of the robot. This could have severely limited the prospect of large sample sizes because of the low numbers of social robots available. Moreover, researchers may not have been able to create and deploy sophisticated health, well-being, or psychological interventions to the same standard as other digital programs onto the robot because of the current software or hardware constraints.

### Opportunities for Further Applications

There were good reasons why the initial studies on social robots have focused on the above participant groups. The NAO robot that has been used in child health is a similar size to a young child and has movements and features that children have generally regarded as acceptable [72], in addition to receiving high acceptability ratings for its application to treatments [73]. Children with ASD tend to have difficulties with human interaction and appropriate expression of emotion, and the robots that have been trialled in that context offer simplified versions of both social interactions and emotional expression that make them particularly suitable for this group [74,75]. The PARO robots used in older age groups are similar in appearance, texture, and behavior to a small, friendly animal and imitate animal-assisted therapy, which is already an established approach for dementia [76], without presenting physical health risks. Thus, social robots in these contexts build on well-established theory and research applications or provide a digital spin to current treatment practice. However, it remains surprising that health-related interventions by social robots do not appear to have been trialled in other problem domains or in adolescents or young adults. Such trials would be timely and important.

Advances in technology equip robots with the capability to conduct health care–related tasks in fields beyond the scope of children, ASD, and elderly care applications. For example, robot capability to respond to information and deliver a structured conversational exchange related to a health care service. These capabilities could translate into health tasks to support patients during their visit, such as verbal discussion of an appropriate homework task, provision of health education during a consultation time, or disclosure of sensitive medical information. Traditional therapeutic elements can also be performed by a robot using these techniques, such as positive verbal affirmations, and providing customized coaching based on interpreting and responding to multiple signal inputs, such automated physiological recordings, self-reported data, or verbal reports on the participant’s progress [77]. Other applications may include encouragement and coaching support for Web-based or digital interventions to increase adherence and impact, as a partial or full substitute for human coaching, which has increased adherence and produced stronger treatment effects in some trials [15]. Robots have already demonstrated an ability to incorporate physical monitoring and service alerts [50], which could in the future reduce response times and hospitalization rates and improve outcomes. Integration of health service robots with personal robots and other digital devices could enhance the transfer of within-session gains to the natural environment, through additional verbal and enactive rehearsal and cueing of behaviors at times in which they are most needed.

Further technological advancements could also progress social robot capabilities beyond limited and constrained tasks. For instance, the use of natural language processing can capture and interpret key elements of human speech [78], and computer vision can recognize faces and activities and detect changes in physiological arousal and emotions [79,80]. Advances in robotic technology are also likely to encompass greater ease of use by nontechnical experts and improved reliability, robustness, and autonomy. Ultimately, these improvements will obviate the need for teleoperation or monitoring by a health professional or trained staff member, which currently occurs in Wizard-of-Oz–style studies, where the role of the robot currently exceeds its technical ability for autonomous operation. Enhancements will also improve the robots’ capacity to deal with uncertain or unpredictable factors in treatment sessions, such as modifying the content of responses and the focus of the session, based on new information from the participant or making a timely response to verbal or nonverbal cues that might indicate disinterest, uneasiness, or annoyance. Such advancements would more accurately emulate the nature of evidence-based face-to-face treatments.

Advances of this kind may be seen by some therapists as a threat. However, we argue that they are more likely to provide exciting opportunities to supplement and augment the impact of standard treatment, reducing time on routine and low-intensity tasks, including information provision, standardized assessment, and treatments that are effective when applied through a highly specified protocol, and increasing the retention and generalization of insights or skills that have been gained within sessions. Practitioners may then focus on more personally satisfying and challenging work, including their relationship with the client; enhancing and maintaining motivation; collaborative goal setting and planning; and addressing severe, complex, or co-occurring problems. However, this proposed outcome remains dependent on continued efforts to develop and test digital health interventions that can be reliably and safely delivered by social robots as their hardware and software progressively increases in sophistication. Although these developments will raise ethical and practical issues and will require careful monitoring of any negative outcomes or potential risks, their benefits to clients and to the cost-effectiveness of health services may be substantial.

### Conclusions

The evidence for health, well-being, and psychosocial interventions that are delivered by social robots remains at an early stage, with few trials being identified. The methodological quality in many trials was reduced by their small sample sizes, an absence of independent randomization, blind assessment or follow-up, and their somewhat rudimentary statistical analyses. However, the higher quality of some recent trials gives cause for optimism that some current and emerging trials will meet more rigorous methodological standards and will steadily move from a focus on efficacy to examining effectiveness in routine care. Progressive reductions in the cost of social robots and improvements in their accessibility for purchase will also make it easier to conduct trials, as will the further development of standard program routines that enable their intuitive and flexible use.

Overall, the initial evidence from clinical trials is promising but is not universally positive. Importantly, some studies show some increases in negative affect or psychiatric symptoms, suggesting that their use with some patient groups may not be indicated or may need more preparation before an intervention is attempted. As yet, there is no evidence that treatment gains from these interventions can be sustained over a follow-up period of more than 3 months or (apart from some trials in aged care facilities) that these interventions can be taken to scale. Nor, as yet, is there evidence on the effects of psychosocial interventions by robots on health and well-being outside the 3 contexts reviewed in this paper.

Currently, trials have used robots with limited capabilities (eg, the PARO fur seal), programmed a robot such as NAO with limited or no branching, or have used Wizard-of-Oz control. None of these approaches fully capitalizes on the potential that robots may have for improving the cost-effectiveness and reach of clinical services, once further development of their hardware and software provides more advanced and reliable social capability.

Each of these limitations is likely to be addressed over the coming years. The true potential for social robots to improve the impact, reach, and cost-effectiveness of health care will then be much clearer than at present.

## Abbreviations

ASD: autism spectrum disorder
COPD: chronic obstructive pulmonary disease
RCT: randomized controlled trial
